# A Wide Dynamic Range RF Attenuation Calibration System for 9 kHz to 10 MHz

**DOI:** 10.3390/s26144379

**Published:** 2026-07-10

**Authors:** Anton Widarta

**Affiliations:** Research Institute for Physical Measurement, National Metrology Institute of Japan, National Institute of Advanced Industrial Science and Technology, Tsukuba 305-8563, Japan; anton-widarta@aist.go.jp; Tel.: +81-50-3521-0882

**Keywords:** RF attenuation calibration, working standard, measurement traceability, RF metrology, direct RF substitution, uncertainty evaluation, electromagnetic compatibility (EMC), wide dynamic range, RF sensing, attenuation standard

## Abstract

Accurate and traceable attenuation measurements are essential for RF instrumentation, communication equipment, electromagnetic compatibility (EMC) testing, and wide dynamic-range signal characterization. This paper presents a practical, accurate, and robust working-standard attenuation measurement system covering the frequency range from 9 kHz to 10 MHz for traceable RF attenuation calibration and wide dynamic-range characterization. The system is based on a direct RF substitution technique and employs two cascaded resistive step attenuators with 10 dB and 1 dB step sizes as the reference standard, providing a total attenuation range of 60 dB with 1 dB resolution. A general-purpose receiver is used as a precision level detector, enabling a simple and fully automated measurement configuration. Traceability is established through calibration against an inductive voltage divider (IVD)-based primary attenuation standard. Owing to the excellent frequency flatness of the reference standard, a single-frequency calibration at 1 MHz is sufficient to characterize the entire operating range from 9 kHz to 10 MHz. The direct measurement capability extends to 60 dB and is further expanded beyond 100 dB through a double-step measurement technique. A comprehensive uncertainty evaluation in accordance with the Guide to the Expression of Uncertainty in Measurement (GUM) yields expanded uncertainties of 3.6 × 10^−3^ dB at 20 dB, 5.6 × 10^−3^ dB at 60 dB, and 8.4 × 10^−3^ dB at 100 dB. Measurements up to 60 dB show excellent agreement with the primary attenuation standard, while alternative validation demonstrates consistency at 80 dB and 100 dB. The obtained uncertainty levels are comparable to or lower than those reported for similar systems in the literature. The proposed system provides a practical and traceable solution for routine RF attenuation calibration, supporting EMC testing, communication-system characterization, RF sensing and measurement applications, and the dissemination of RF metrological traceability.

## 1. Introduction

Accurate control and measurement of signal amplitudes are essential for ensuring linearity and wide dynamic range performance in radio frequency (RF) systems, particularly in applications requiring traceable RF calibration and accurate dynamic range characterization [1,2,3,4,5]. In RF-based sensing and measurement systems, signal amplitude directly affects measurement accuracy, data integrity, and overall system performance. Traceable attenuation standards are therefore fundamental for enabling consistent and comparable measurements across applications such as RF instrumentation, communication equipment, radar systems, and electromagnetic compatibility (EMC) testing [6,7,8,9,10]. These capabilities are essential for ensuring the reliability and traceability of sensor-related measurements in RF sensing and instrumentation applications.

While most attenuation standards developed at National Metrology Institutes (NMIs) focus on frequencies above several tens of megahertz, spanning the microwave to millimeter-wave frequency regions [11,12,13,14,15,16,17,18,19,20,21,22], there has been increasing demand in recent years for traceable attenuation calibration at frequencies below 10 MHz, particularly starting from 9 kHz. This demand is driven by applications such as EMC instrumentation, RF measurement systems, and the wide dynamic range characterization of communication devices [23,24]. Therefore, practical and traceable calibration systems covering the frequency range from 9 kHz to 10 MHz are strongly needed, especially those offering high accuracy, wide dynamic range, and operational simplicity. However, only a limited number of such standard systems have been developed so far [25,26,27].

In previous work, a primary attenuation standard measurement system covering the frequency range from 1 kHz to 10 MHz was developed based on an inductive voltage divider (IVD)-referenced null-detection technique [28]. This approach provides high accuracy and well-characterized uncertainty; however, it involves relatively complex measurement procedures, including null balancing, heterodyne down-conversion, and specialized configurations required to maintain accuracy over a wide attenuation range. In that implementation, the achievable attenuation was limited to approximately 60 dB. Consequently, although suitable as a primary standard, it is not well suited for routine calibration or efficient dissemination of traceability, particularly at higher attenuation levels.

To address this need, a practical and robust working-standard attenuation measurement system is presented for traceable, wide dynamic-range RF attenuation calibration and characterization beyond 100 dB over the frequency range from 9 kHz to 10 MHz. The system builds on the feasibility demonstrated in earlier studies [29], with a focus on experimental uncertainty analysis and the implementation of a double-step measurement technique [30] to extend the measurable attenuation range. The system is based on a direct RF substitution technique with a simple configuration, eliminating the need for frequency conversion. A resistive step attenuator calibrated at 1 MHz is employed as a reference standard over the entire frequency range, while a general-purpose receiver serves as a precision level detector. The system enables fully automated operation, thereby simplifying routine calibration and enhancing measurement stability. A comprehensive uncertainty evaluation, including both Type A and Type B contributions, is conducted to ensure reliable and traceable measurement results. Such capability is increasingly important for RF instrumentation, EMC measurements, communication-system characterization, and RF sensing and measurement applications, while supporting the dissemination of RF metrological traceability.

## 2. System Configuration and Measurement Principle

### 2.1. Measurement System

**Figure 1:**
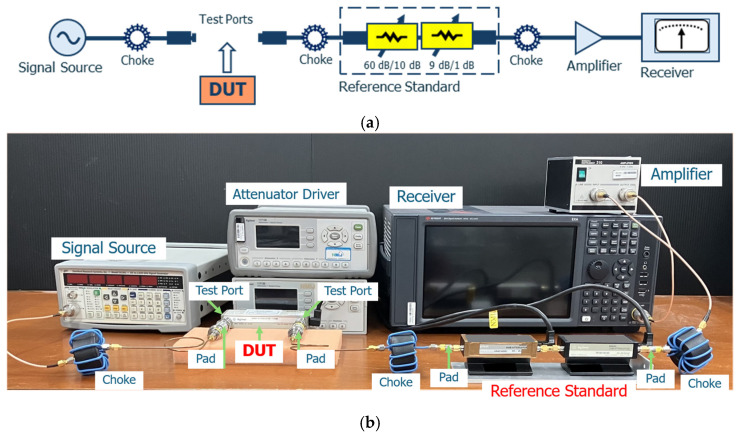
(**a**) Block diagram and (**b**) photograph of the proposed working standard attenuation measurement system in the frequency range from 9 kHz to 10 MHz, comprising an RF signal source, test ports, a resistive step-attenuator reference (10 dB and 1 dB steps), low-noise amplifier and a general purpose receiver; the DUT is inserted between the test ports.

Figure 1 illustrates (a) a simplified block diagram and (b) a photograph of the proposed working-standard attenuation calibration system covering the frequency range from 9 kHz to 10 MHz, based on a direct RF substitution technique without the need for frequency conversion. The system is designed with a simple and robust configuration, comprising an RF signal source (Model SG382, Stanford Research Systems, Sunnyvale, CA, USA), test ports, a 60 dB reference standard with 1 dB resolution realized using two programmable step attenuators (Model 84906K, Keysight Technologies, Santa Rosa, CA, USA with 10 dB steps and Model 84904K, Keysight Technologies, Santa Rosa, CA, USA with 1 dB steps) operated by an attenuator switch driver (Model 11713B, Keysight Technologies, Santa Rosa, CA, USA), a 30 dB gain low-noise amplifier (LNA, Model 310, Sonoma Scientific Inc., Santa Rosa, CA, USA), and a general-purpose receiver (Model N9010B, Keysight Technologies, Santa Rosa, CA, USA) serving as a precision level detector. The reference standard is described in detail in the next subsection. Each test port incorporates a 10 dB matching pad (Model 8491B, Keysight Technologies, Santa Rosa, CA, USA), for type-N connectors or Model 8493C, Keysight Technologies, Santa Rosa, CA, USA, for 3.5 mm connectors) together with a toroidal ferrite choke. The matching pads are used to reduce impedance mismatch effects, thereby minimizing mismatch-related uncertainties. Meanwhile, the toroidal ferrite chokes suppress leakage currents, which become significant at low frequencies because of reduced shielding effectiveness associated with the skin effect in coaxial transmission lines [28]. Additional toroidal ferrite chokes are strategically placed at critical points in the system, including the input and output of the reference standard, to further suppress unwanted leakage and improve measurement stability. The device under test (DUT), which may generally be a fixed, variable, or step attenuator, is inserted between the test ports. In the present work, fixed (non-tunable) resistive step attenuators are used.

The attenuation of the DUT is determined by comparison with the reference standard, with the receiver operating as a sensitive signal comparator. During measurement, the power level difference between the initial and final signals applied to the receiver is maintained within 1 dB by appropriate adjustment of the reference standard before and after insertion of the DUT, thereby minimizing the range-dependent error of the receiver. Let *S* (dB) denote the calibrated attenuation value of the reference standard, and let $D_{i}$ (dBm) and $D_{f}$ (dBm) represent the receiver readings before and after inserting the DUT, respectively. The attenuation A (dB) of the DUT is then determined as:

(1) $$\begin{array}{cccc} & A=S+D_{i}-D_{f}. & & \end{array}$$

A personal computer is used to automate the measurement process, including control of the RF signal source (frequency and power level), adjustment of the reference attenuator settings, acquisition of receiver readings, and calculation of the attenuation according to Equation (1). For programmable variable or step attenuator calibration, the entire procedure can be performed automatically.

### 2.2. Reference Standard

**Figure 2:**
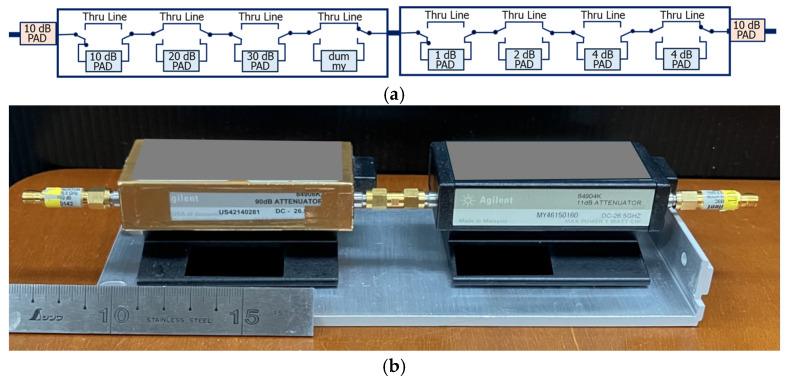
Reference standard assembly using cascaded resistive step attenuators (10 dB and 1 dB steps), showing (**a**) internal configuration and (**b**) external implementation.

The reference standard is realized by cascading two programmable resistive step attenuators, namely a 10 dB step attenuator (Model 84906K, Keysight Technologies, Santa Rosa, CA, USA) and a 1 dB step attenuator (Model 84904K, Keysight Technologies, Santa Rosa, CA, USA), operated using an attenuator switch driver (Model 11713B, Keysight Technologies, Santa Rosa, CA, USA), thereby providing a total attenuation range of 60 dB with 1 dB resolution. Figure 2 shows both a schematic diagram of the internal configuration (a) and a photograph of the external assembly (b), including the step attenuator unit, attached 10 dB matching pads (Model 8493C, Keysight Technologies, Santa Rosa, CA, USA), and the aluminum-alloy base. The attenuators are mounted on an aluminum-alloy base to enhance mechanical stability and robustness. Traceability of the reference standard is ensured through calibration against the IVD-based primary attenuation standard [28]. The matching pads preserve the calibrated attenuation values by minimizing impedance mismatch effects when the attenuators are used as the reference standard in the working standard system.

Owing to the inherently flat frequency response of this type of precision RF resistive attenuator, a single-frequency calibration at 1 MHz is sufficient to characterize its attenuation over the entire operating range from 9 kHz to 10 MHz. Furthermore, since the reflection coefficients of the individual attenuators are negligible within this frequency range, additional matching pads between them are unnecessary. Consequently, independent calibration of each attenuator is sufficient, allowing intermediate attenuation values to be accurately determined by summing the calibrated step values, without requiring direct calibration of their combined attenuation. This approach reduces the number of required calibration points from 60 to 15, significantly improving the efficiency of the traceability process. Eliminating these additional matching pads also reduces the insertion loss of the reference path, thereby improving the dynamic range.

### 2.3. Double-Step Measurement Technique

**Figure 3:**
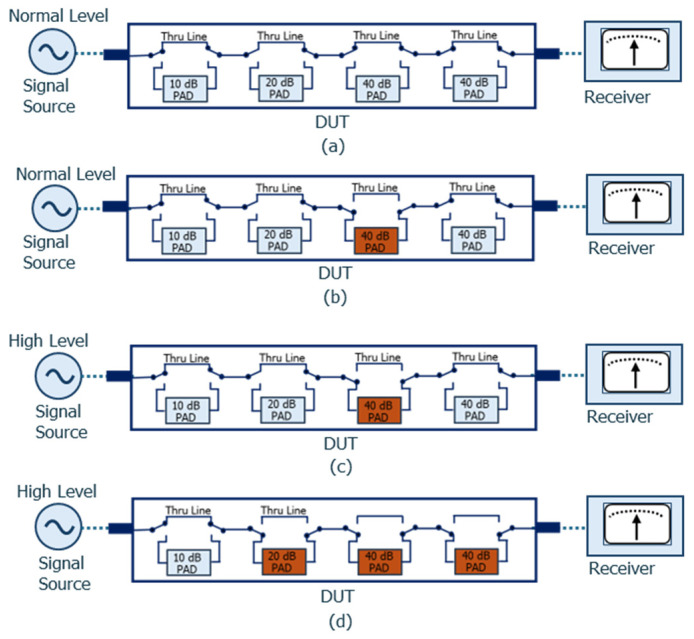
Measurement configurations used in the double-step technique: (**a**) through connection; (**b**) 40 dB module (No. 3) configured as a gauge block attenuator (GBA); (**c**) GBA configuration with increased output power; and (**d**) DUT configuration with nominal 100 dB attenuation realized by combining modules No. 2, No. 3, and No. 4.

The measurement capability of attenuation measurement systems is fundamentally limited by the linearity, or dynamic range, of the detectors or receivers employed, defined as the difference between the compression power level and the noise floor of the receiver. To overcome this limitation, a double-step measurement technique is employed in this work to enable the measurement of attenuation levels exceeding 60 dB. This technique has been described in a previous study [30]; for completeness, its application to the calibration of a step attenuator with a nominal attenuation of 100 dB is briefly reviewed below as an example. As shown in Figure 3, a nominal attenuation of 100 dB of the DUT is measured using the proposed system in conjunction with the double-step technique. The step attenuator consists of four modules: No. 1 (10 dB), No. 2 (20 dB), No. 3 (40 dB), and No. 4 (40 dB). The nominal 100 dB attenuation is realized by combining modules No. 2, No. 3, and No. 4.

In Step 1, the attenuation between the through connection (Figure 3a) and the active 40 dB module (No. 3), configured as a gauge block attenuator (GBA) in the signal path (Figure 3b), is measured. In Step 2, the output power of the RF signal source is appropriately increased, and the attenuation between the active GBA (Figure 3c) and the DUT set to 100 dB is measured. The GBA reduces the increased power level, thereby preventing overloading or saturation of the LNA and the receiver. The attenuation of the DUT is then obtained by summing the results of these two measurement steps.

## 3. Measurement Results and Uncertainty Evaluation

This section provides a comprehensive evaluation of the measurement uncertainty associated with the proposed working-standard attenuation system. All measurements and analyses were performed under controlled laboratory conditions (temperature: 23 ± 1 °C; relative humidity: 50% ± 20%). The non-statistical (Type B) uncertainty contributions are grouped into three principal categories: (i) uncertainties related to the assembled reference standard; (ii) system-related effects, including nonlinearity, stability, resolution, and impedance mismatch; and (iii) contributions associated with the gauge block attenuator (GBA), which are further examined in relation to the double-step measurement approach. All uncertainty components are evaluated following the framework of the Guide to the Expression of Uncertainty in Measurement (GUM) [31,32].

(1) Reference Standard

*a*. *Calibration:*

The reference standard, formed by two step attenuators, was calibrated at 1 MHz against the primary attenuation standard based on the voltage ratio of the IVD [28]. The first attenuator was calibrated in 10 dB steps up to 60 dB. The corresponding standard uncertainty $u(X_{1a})$ is 1.1 × 10^−3^ dB for attenuation values up to 20 dB, 1.3 × 10^−3^ dB for values up to 40 dB, and 1.9 × 10^−3^ dB for values up to 60 dB, obtained from the expanded uncertainty stated in the calibration certificate by dividing by the coverage factor *k* = 2. Similarly, the second attenuator was calibrated in 1 dB steps up to 9 dB, with a standard uncertainty of 1.1 × 10^−3^ dB. Intermediate attenuation values are obtained by summing the calibrated step values of the two attenuators. For example, a nominal attenuation of 43 dB is realized by summing 40 dB from the first attenuator and 3 dB from the second attenuator. Accordingly, the standard uncertainty of an intermediate attenuation value obtained from the combination of two calibrated steps can be evaluated using the root-sum-square (RSS) method. In this case, the standard uncertainty $u(X_{1a})$ for a nominal attenuation of 43 dB is evaluated as 1.7 × 10^−3^ dB, obtained from the RSS combination of the individual step uncertainties.

**Figure 4:**
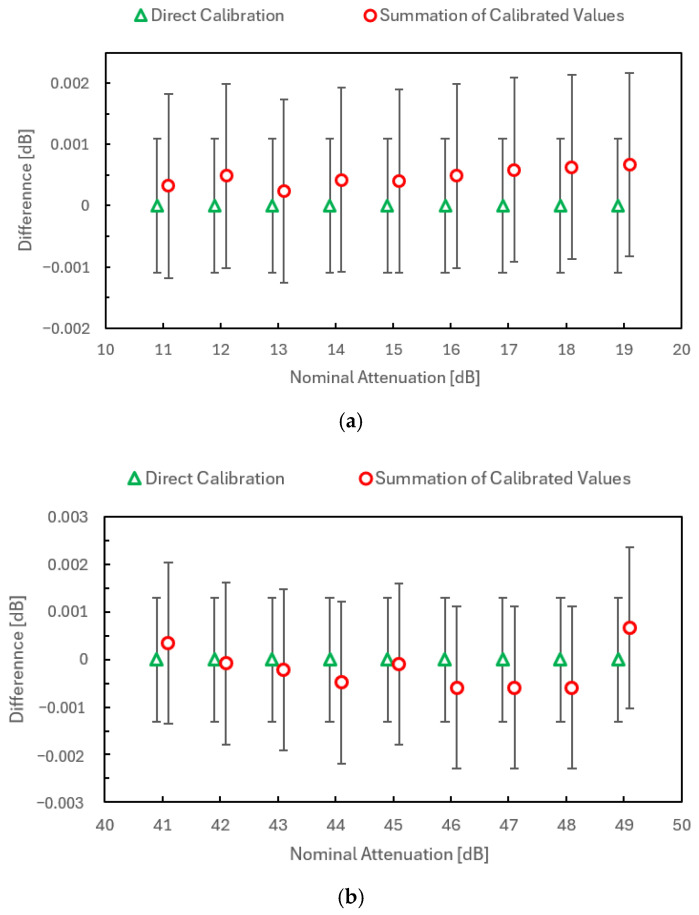
Comparison of intermediate attenuation values obtained using the summation of calibrated step values and direct measurements: (**a**) nominal values from 11 dB to 19 dB; (**b**) nominal values from 41 dB to 49 dB. Circles represent the summed calibrated values, while triangles represent the direct calibration results. Error bars indicate the expanded uncertainty. The data are normalized to the direct calibration results.

To validate the intermediate attenuation values, attenuation values from 11 dB to 19 dB and from 41 dB to 49 dB were evaluated in 1 dB steps using both direct calibration and the proposed summation method. The results, shown in Figure 4a,b, demonstrate excellent agreement, with differences well within the expanded uncertainties; circles represent the proposed summation method, whereas triangles denote direct calibration. This confirms the validity of both the intermediate value determination method and the associated standard uncertainties.

*b*. *Frequency Dependence:*

The frequency dependence of the reference standard was evaluated over the full operating frequency range of 9 kHz to 10 MHz using the primary attenuation standard system [28], with attenuation levels from 10 dB to 60 dB in 10 dB increments. The maximum measured deviations, normalized to the results at 1 MHz, were 1.0 × 10^−3^ dB, 2.0 × 10^−3^ dB, and 3.0 × 10^−3^ dB for attenuation levels of 20 dB, 40 dB, and 60 dB, respectively. All deviation remained within the corresponding expanded uncertainty of 2.2 × 10^−3^ dB, 2.6 × 10^−3^ dB, and 3.8 × 10^−3^ dB. These results confirm that a single-frequency calibration at 1 MHz is sufficient to characterize the attenuation over the entire frequency range. Therefore, the standard uncertainties due to frequency dependence $u(X_{1b})$ are estimated to be 2.9 × 10^−4^ dB, 5.8 × 10^−4^ dB, and 8.7 × 10^−4^ dB for the 20 dB, 40 dB, and 60 dB attenuation levels, respectively, calculated as half of the maximum deviation divided by $\sqrt{3}$, assuming a uniform probability distribution.

*c*. *Mismatch:*

In RF measurement systems, impedance mismatches cause multiple reflections between components, leading to interference and deviations in the measured attenuation. The applied equation models this effect by relating the mismatch uncertainty to the reflection coefficients of the source (*Γ*_G_) and load (*Γ*_L_), as well as the S-parameters of the reference standard. The standard uncertainty of the reference standard due to impedance mismatch ($\sigma_{M}$) is then evaluated using the following expression [33].

(2) $$\begin{array}{c} \sigma_{M}=\frac{8.686}{\sqrt{2}}{\left\{{\left|\Gamma_{G}\right|}^{2}{\left|S_{11i}-S_{11f}\right|}^{2}\;+\;{\left|\Gamma_{L}\right|}^{2}\left|S_{22i}-S_{22f}\right|\right.}^{2}\\ {\left.+{\left|\Gamma_{G}\right|}^{2}{\left|\Gamma_{L}\right|}^{2}{\left|S_{21i}^{2}-S_{21f}^{2}\right|}^{2}\right\}}^{1/2}. \end{array}$$

Here, *S_ij_* denotes the S-parameters of the reference standard, where the subscripts *i* and *f* represent the initial and final states, respectively. The S-parameters were measured using a vector network analyzer (VNA) over all attenuation settings across the entire frequency range. The measured values satisfy |*S_ii_*| ≤ 0.01, indicating a negligible contribution to the mismatch uncertainty. The source reflection coefficient, *Γ_G_*_,_ is determined from|*S*_22_| of the 10 dB matching pad at the second test port and is found to be less than or equal to 0.005. In contrast, the load reflection coefficient, *Γ_L_*_,_ corresponds to that of the amplifier, for which the manufacturer specifies a value of 0.3. This relatively large magnitude constitutes a primary contributor to mismatch uncertainty. Due to the inclusion of 10 dB matching pads in the reference standard, both *Γ_G_* and *Γ_L_* are reduced by approximately a factor of ten, resulting in *Γ_G_ ≈* 0.0005 and *Γ_L_ ≈* 0.03. Substituting these values into Equation (2), the mismatch uncertainty $u(X_{1c})$ of the reference standard during system operation is estimated to be less than 5.0 × 10^−4^ dB.

(2) System Nonlinearity

**Figure 5:**
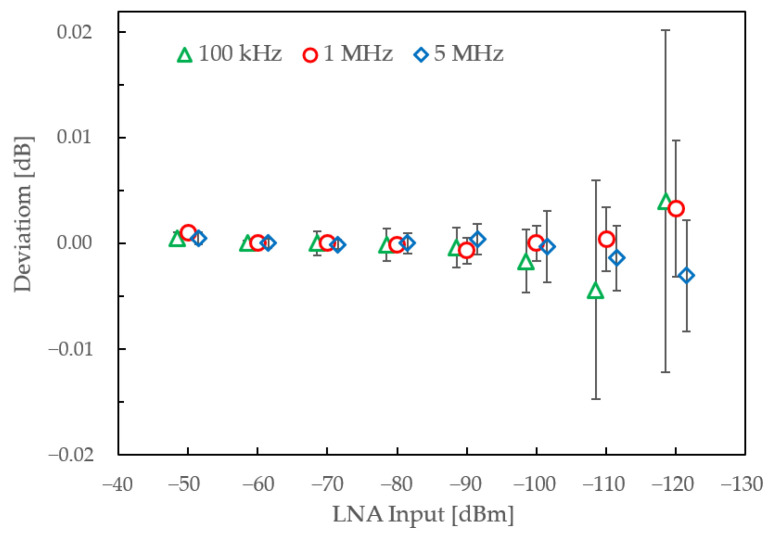
Linearity of the proposed attenuation measurement system at 100 kHz, 1 MHz and 5 MHz.

The measurable attenuation range, or dynamic range, of the system is primarily determined by its linearity characteristics. To evaluate these characteristics, experiments were conducted to assess the overall system linearity, including contributions from saturation, leakage, and noise. Figure 5 presents the measurement results obtained at 100 kHz, 1 MHz, and 5 MHz by applying 10 dB attenuation increments using a variable step attenuator as the DUT. The input power level to the LNA was varied in 10 dB steps, starting from −50 dBm, with the RF source output level set to 0 dBm and the DUT configured for 10 dB attenuation. The receiver was configured as follows to achieve optimal accuracy: frequency span of 0 Hz, bandwidth of 100 Hz, and averaging of 50. Each measurement point was further averaged over five repetitions to enhance measurement stability. All measured attenuation values were normalized with respect to the measurement at −60 dBm. The horizontal axis represents the input power level to the LNA, while the vertical axis shows the deviation from the normalized attenuation referenced to −60 dBm. The error bars represent the standard deviation of the measurements, which is incorporated into the system noise and treated as a Type A (random) uncertainty component, as described below. For visualization purposes, the error bars at LNA input levels of −110 dBm and −120 dBm are scaled to U/2 and U/4, respectively. A systematic frequency-dependent trend is observed, reflecting underlying measurement system effects. In particular, at LNA input levels of −110 dBm and below, significantly larger deviations are observed at 100 kHz compared to those at MHz frequencies. This behavior is attributed to the increased influence of flicker noise at lower frequencies, where it becomes dominant, in contrast to higher frequencies where white noise prevails.

The system exhibits strong linearity performance, with deviations remaining below 0.002 dB and a standard deviation of approximately 0.003 dB even at LNA input levels down to −100 dBm. The maximum observed nonlinearities were 6.4 × 10^−4^ dB for attenuation levels up to 40 dB (−80 dBm) and 1.5 × 10^−3^ dB for levels up to 60 dB (−100 dBm). For attenuation levels beyond 60 dB, a double-step measurement approach is applied, allowing the RF source power to be increased to 20 dBm or higher while avoiding saturation of both the LNA and the receiver. Consequently, the nonlinearity are 1.5 × 10^−3^ dB for measurements up to 80 dB (−100 dBm) and 3.5 × 10^−3^ dB for measurements up to 100 dB (−120 dBm). The corresponding standard uncertainties $u(X_{2})$ are calculated as half of the maximum deviation mentioned above divided by $\sqrt{3}$, assuming a uniform probability distribution.

(3) Stability

The stability of the system can be classified into short-term stability, representing system drift, and long-term stability. To mitigate the effect of drift, the initial (reference) measurement is performed twice for each measurement point—before and after the calibration point—and the average value is taken. The drift is observed for less than 0.002 dB. The corresponding standard uncertainties $u(X_{3a})$ are calculated as half of the maximum deviation divided by $\sqrt{3}$, assuming a uniform probability distribution.

The long-term stability is assessed by analyzing calibration data of a step attenuator employed as the DUT over a three-year measurement period. The corresponding standard uncertainties $u\left(X_{3b}\right),$ were obtained as 6.5 × 10^−4^ dB, 7.1 × 10^−4^ dB, 1.1 × 10^−3^ dB, 1.2 × 10^−3^ dB, and 2.1 × 10^−3^ dB, for attenuation levels of 20 dB, 40 dB, 60 dB, 80 dB and 100 dB, respectively.

(4) Digital Reading Resolution

The digital resolution of the receiver is 0.001 dB. The corresponding standard uncertainty $u(X_{4})$, is evaluated as 2.9 × 10^−4^ dB, assuming a rectangular distribution, where the uncertainty is given by half of the resolution divided by $\sqrt{3}$.

(5) Mismatch

Each test port uses a precision 10 dB pad to reduce mismatch, yielding source and load reflection coefficients (*Γ_G_*, *Γ_L_*) below 0.01. The DUT also shows good matching performance in this frequency range (∣*S_ii_*∣ ≤ 0.01). Using Equation (2), the mismatch uncertainty $u(X_{5})$, is estimated as 1.0 × 10^−3^ dB under a U-shaped distribution.

(6) Gauge Block Attenuator (GBA)

As described in Section 2.3, for measurements exceeding 60 dB, a double-step technique is employed, in which the GBA (40 dB in this study) is first measured at a normal power level using the single-step technique. In the second step, the DUT with high attenuation, including the GBA, is calibrated at a higher power level. The standard uncertainty, $u(X_{6})$, associated with the GBA is evaluated from single-step measurements performed within the same system.

(7) Standard Deviation of the Mean

The Type A standard uncertainty, $u(X_{7})$, is evaluated as the standard deviation of the mean (SDOM) from 10 repeated measurements. Thus, $u(X_{7})=s/\sqrt{10}$, where *s* denotes the standard deviation of the measurement results.

Table 1 provides a summary of the estimated uncertainties for attenuation measurements of a step attenuator. The results are presented for nominal attenuation levels of 20 dB, 40 dB, and 60 dB using the single-step measurement method, and for 80 dB and 100 dB using the double-step technique, across the frequency range from 9 kHz to 10 MHz. The expanded uncertainty is obtained by multiplying the combined standard uncertainty by a coverage factor of *k* = 2, corresponding to an approximate 95% confidence level under the assumption of a normal distribution. The resulting uncertainty values are comparable to those of the primary attenuation standard, specifically 2.2 × 10^−3^ dB at 20 dB, 2.6 × 10^−3^ dB at 40 dB, and 3.8 × 10^−3^ dB at 60 dB, and are consistent with or lower than values reported for primary standards in other NMIs [25,26,27].

## 4. System Verification

**Figure 6:**
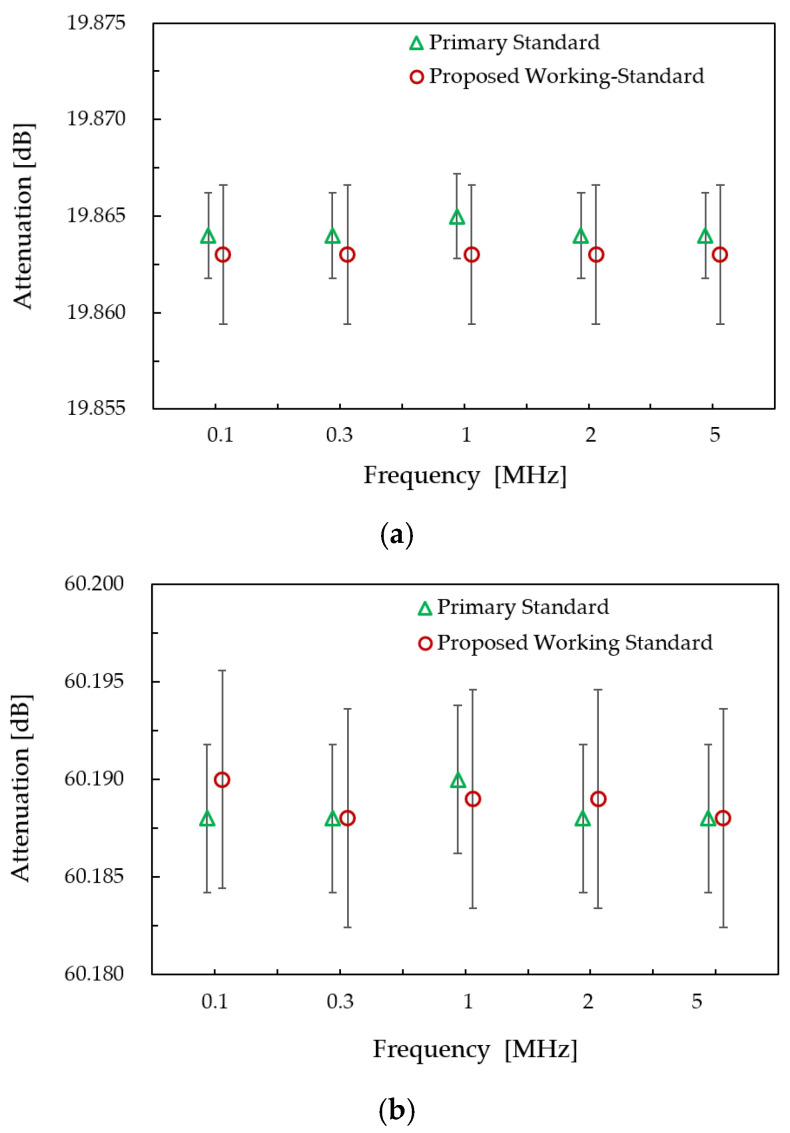
Verification of the proposed measurement system against the primary attenuation standard: (**a**) 20 dB attenuation measured at 100 kHz, 300 kHz, 1 MHz, 2 MHz, and 5 MHz; (**b**) 60 dB attenuation measured over the same frequency range.

The effectiveness of the proposed system was assessed by measuring RF resistive step attenuators (Model 84906L, Keysight Technologies, Santa Rosa, CA, USA) with nominal values of 20 dB and 60 dB, and (Model 8496H, Keysight Technologies, Santa Rosa, CA, USA) with nominal values of 80 dB and 100 dB over the frequency range from 100 kHz to 5 MHz. Measurements for the 20 dB and 60 dB attenuators were compared with reference results obtained using the primary attenuation standard [28]. The comparison is presented in Figure 6a,b, where circles represent the proposed working standard and triangles denote the primary attenuation standard. Each reported value corresponds to the mean of ten repeated measurements, with vertical error bars indicating the expanded uncertainties. The two systems show good agreement. A slight spike is observed around 1 MHz in the results obtained using the primary standard, which may be attributed to frequency-dependent effects, including the characteristics of the DUT’s resistive elements and impedance mismatch. Nevertheless, the deviation remains within the expanded uncertainty limits and does not affect the overall validity of the results. This agreement was further evaluated using the normalized error, En, for which all obtained |En| values were less than 1, confirming consistency within the expanded uncertainties. Moreover, the |En| values were below 0.5, indicating a high level of agreement and supporting the validity of the uncertainty evaluation.

**Figure 7:**
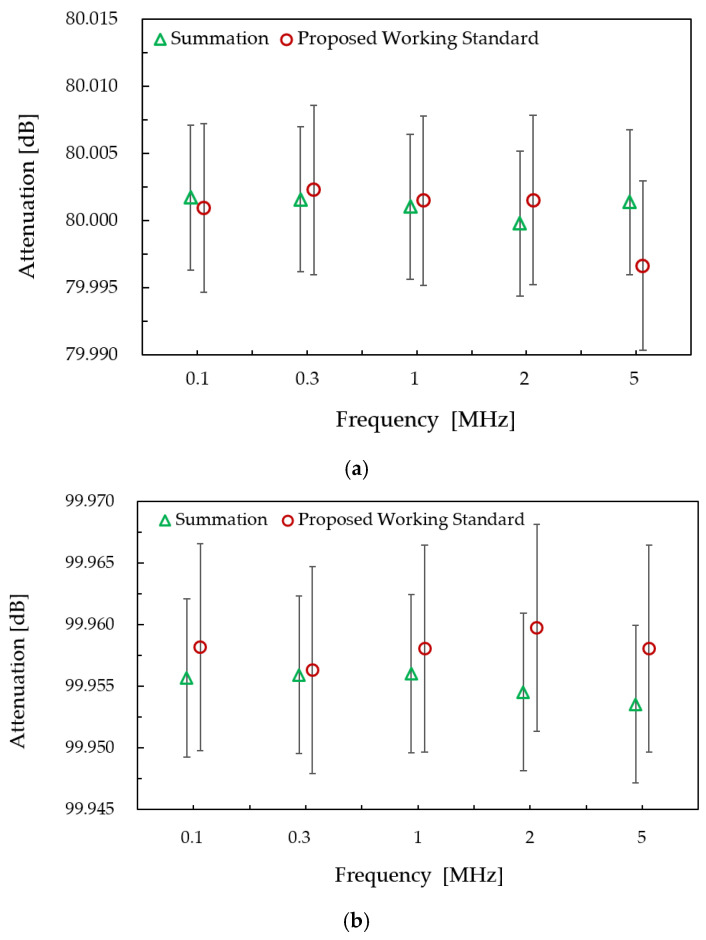
Verification of the proposed measurement system against the estimated attenuation values obtained by summing the individually measured attenuators: (**a**) 80 dB attenuation measured at 100 kHz, 300 kHz, 1 MHz, 2 MHz, and 5 MHz; (**b**) 100 dB attenuation measured over the same frequency range.

For the 80 dB and 100 dB measurements, direct comparison with the primary attenuation standard is not feasible due to its limited measurement capability [28]. However, as described in Section 2.2, the reflection coefficients of the individual attenuators of this step attenuator type, which contribute to mismatch uncertainties, are negligible within the frequency range of interest. Therefore, the total attenuation may be estimated by summing the individually measured values of each attenuator, and the expanded uncertainties are approximately derived from the uncertainties of the individual modules using RSS method. These values are used for comparison purposes only and do not constitute a direct validation against the primary attenuation standard. The results obtained from the proposed system are then compared with these estimated values as an alternative validation, as shown in Figure 7a,b. The results show good agreement, with all observed differences remaining within both uncertainty limits. However, a relatively noticeable deviation is observed, particularly at 2 MHz and 5 MHz. This deviation may be attributed to potential crosstalk between modules and impedance mismatch, whose effects are not fully accounted for in the estimated values obtained by summing the individual module measurements. Further validation will be pursued through bilateral comparisons with other NMIs and participation in international key comparisons under the framework of the International Bureau of Weights and Measures (BIPM) [27].

## 5. Conclusions

This paper has presented a practical and robust working-standard attenuation measurement system for traceable RF attenuation calibration and wide dynamic-range characterization from 9 kHz to 10 MHz. The system is based on a direct RF substitution technique and employs two cascaded resistive step attenuators as the reference standard. Traceability is established through calibration against the primary attenuation standard. The excellent frequency flatness of the attenuators enables characterization of the entire frequency range through single-frequency calibration, while their negligible mismatch allows attenuation values to be established efficiently from individually calibrated attenuators. A further advantage of the proposed approach is the extension of the measurable attenuation range beyond 100 dB through the double-step measurement technique while preserving good linearity and measurement stability. Uncertainty analysis and experimental validation demonstrate the suitability of the system for traceable attenuation calibration. Measurements up to 60 dB show excellent agreement with the primary attenuation standard, while alternative validation supports the consistency of measurements at 80 dB and 100 dB. The proposed system provides an efficient means of disseminating attenuation traceability from the primary standard to practical calibration services. Its simple configuration, automation capability, and wide dynamic range make it well suited for routine RF attenuation calibration and support a broad range of applications, including EMC measurements, communication-system characterization, and RF sensing and measurement systems requiring accurate and traceable signal characterization.

## Figures and Tables

**Table 1:** Summary of the uncertainty estimation for attenuation measurements.

Uncertainty Source		Category	Prob. Dis.	Nominal Attenuation [dB]				
				20	40	60	80	100
				*u*(*X_i_*) [dB]	*u*(*X_i_*) [dB]	*u*(*X_i_*) [dB]	*u*(*X_i_*) [dB]	*u*(*X_i_*) [dB]
1	Reference Standard							
	1a Calibration	B	Unif.	1.1 × 10^−3^	1.3 × 10^−3^	1.9 × 10^−3^	1.3 × 10^−3^	1.9 × 10^−3^
	1b Frequency Dependence	B	Unif.	2.9 × 10^−4^	2.9 × 10^−4^	8.7 × 10^−4^	8.7 × 10^−4^	8.7 × 10^−4^
	1c Mismatch (Standard)	B	U	5.0 × 10^−4^	5.0 × 10^−4^	5.0 × 10^−4^	5.0 × 10^−4^	5.0 × 10^−4^
2	Linearity	B	Unif.	1.8 × 10^−4^	1.8 × 10^−4^	4.3 × 10^−4^	4.3 × 10^−4^	1.0 × 10^−3^
3	Stability							
	3a Short Term (drift)	B	Unif.	2.9 × 10^−4^	2.9 × 10^−4^	2.9 × 10^−4^	5.8 × 10^−4^	5.8 × 10^−4^
	3b Long-term	B	Unif.	6.5 × 10^−4^	7.1 × 10^−4^	1.1 × 10^−3^	1.2 × 10^−3^	2.1 × 10^−3^
4	Digital Reading Resolution	B	Unif.	2.9 × 10^−4^	2.9 × 10^−4^	2.9 × 10^−4^	2.9 × 10^−4^	2.9 × 10^−4^
5	Mismatch (DUT)	B	U	1.0 × 10^−3^	1.0 × 10^−3^	1.0 × 10^−3^	1.0 × 10^−3^	1.0 × 10^−3^
6	Gauge Block Attenuator	B	Unif.				1.9 × 10^−3^	1.9 × 10^−3^
7	SDOM	A	Norm.	2.0 × 10^−4^	3.0 × 10^−4^	9.0 × 10^−4^	9.0 × 10^−4^	1.6 × 10^−3^
*u*_c_	Combined Standard Uncertainty			1.8 × 10^−3^	1.9 × 10^−3^	2.8 × 10^−3^	3.2 × 10^−3^	4.2 × 10^−3^
*U*	Expanded Uncertainty			3.6 × 10^−3^	3.8 × 10^−3^	5.6 × 10^−3^	6.4 × 10^−3^	8.4 × 10^−3^

## Data Availability

The data presented in this study are available within the article.

## Abbreviations

The following abbreviations are used in this manuscript:

DUT	Device Under Test
EMC	Electromagnetic Compatibility
GBA	Gauge Block Attenuator
GUM	Guide to the Expression of Uncertainty in Measurement
IVD	Inductive Voltage Divider
LNA	Low Noise Amplifier
NMI	National Metrology Institute
Prob. Dis.	Probability Distribution
RF	Radio Frequency
RSS	Root-Sum-Square
SDOM	Standard Deviation of the Mean
Unif.	Uniform
VNA	Vector Network Analyzer
